# “The most stress comes from witnessing the abuse of children” *—*challenges faced by research assistants in community-based research in Mtwara, Tanzania

**DOI:** 10.1186/s12889-026-26485-3

**Published:** 2026-02-07

**Authors:** Salum Mshamu, Bipin Adhikari, Judith Meta, Salma Halifa, Lorenz von Seidlein, Frédérique Vallières

**Affiliations:** 1 CSK Research Solutions, Mtwara, Tanzania; 2 Centre for Tropical Medicine and Global Health, Nuffield Department of Medicine, University of Oxford, Oxford, UK; 3 Mahidol Oxford Tropical Medicine Research Unit, Faculty of Tropical Medicine, Mahidol University, Bangkok, Thailand; 4 Private Consultant, Social Scientist, Mtwara, Tanzania; 5 Trinity Centre for Global Health, Trinity College Dublin, Dublin, Leinster, Ireland

**Keywords:** Community-based research, Field work, Research assistants, Child sexual abuses, Tanzania, Moral distress

## Abstract

**Background:**

Field workers or research assistants (RAs) are commonly employed within community-based trials. Training provided for RAs, however, is often limited to the technical elements of research, with little-to-no training to help them navigate the ethical challenges they may encounter while working in community-based settings. The main objective of this study was to explore the challenges faced by RAs working as part of a novel housing initiative, to describe the impact these challenges had on their work and wellbeing, and to outline approaches taken by RAs in facing these challenges.

**Methods:**

A qualitative interview guide was piloted and refined for key informant interviews (KIIs) with research assistants (RAs) working on the Star Homes intervention in Mtwara, southern Tanzania. A total of 16 KIIs were conducted with all available RAs. These data were supplemented with 47 documents that comprised 31 case studies and 16 observation notes. The interviews were conducted in Kiswahili, audio-recorded, transcribed verbatim, and translated into English. All transcripts underwent line-by-line coding in NVivo and were analysed using thematic analysis.

**Results:**

Principal challenges included difficulties related to RAs’ roles and responsibilities, including having to travel long distances for data collection. Most prominent, however, were ethical challenges, including witnessing the suffering of study participants. RAs unanimously expressed being most distressed by encountering children who were neglected or subjected to abuse, including several cases of child sexual abuse (CSA), as part of their work.

**Conclusions:**

Clear engagement strategies with communities and authorities are essential to support RAs in reporting and follow-up. Training and resources are urgently needed to prepare field staff, mitigate moral distress, and protect research participants from harmful behaviours.

**Supplementary Information:**

The online version contains supplementary material available at 10.1186/s12889-026-26485-3.

## Background

Global health studies often concern communities facing multidimensional poverty, carrying implications for study design and accompanying community engagement [1, 2]. Poverty, for example, is a risk factor for most infectious diseases, resulting in researchers often targeting high-risk, high-poverty populations as their primary population of interest [1, 3]. To reach high-risk populations, researchers commonly rely on research assistants (RAs), often nationals of the country and sometimes members of the study communities, who are then trained to support research implementation (and less often its design and analysis). Indeed, the integration of RAs is encouraged in global health to enhance community engagement, contextual relevance, and local ownership of research processes [4]. RAs are thus considered essential for facilitating data collection, ensuring recognition of both community and researcher priorities, building participant trust, increasing uptake of research findings, and generally acting as a bridging agent between researchers and communities [5].

Unlike clinical trials conducted in more controlled (e.g., hospital) settings, community-based clinical trials are embedded within the everyday realities of social life. This ‘real world’ dimension implies that such trials interact with the social dynamics of the communities in which they are conducted [6, 7]. RAs are thus active contributors to the social fabric of a study environment and may become entangled in the complex interpersonal dynamics of a community [8], including within households participating in research [9]. The numerous challenges of poverty that shape the daily life of trial participants and wider communities can be hard to ignore [3] and RAs often witness events and behaviours that extend beyond their scope of work. Additionally, RAs often occupy the lower end of the work hierarchy, engage in precarious work [10] on short-term (if any) contracts, and are often poorly remunerated. It is therefore unsurprising that RAs report multiple work-related stressors, ranging from psychosocial distress to adverse physical health [11–13]. Specifically, RAs report experiencing emotional trauma from witnessing poverty, illness, and violence in their communities, leading to secondary traumatic stress and burnout [14, 15].

The extant literature provides a range of recommendations on how to train and build the capacity of RAs on topics such as research ethics [16], including obtaining informed consent [17], protecting participants, and conducting recruitment procedures—particularly when working with historically underserved communities [18]. Guidance also exists on administering surveys, facilitating focus group discussions and engaging in reflective practice [19]. However, and while instances of misconduct or misbehaviour by RAs working with at-risk populations have been documented [20], there remains limited guidance on how RAs should prepare for and manage challenging field situations, including the social and psychological risks that arise when encountering crimes or harms such as child sexual abuse (CSA) perpetrated by participants. Not knowing whether—and if so, how—to respond to such complex ethical and legal challenges can cause significant moral distress for RAs [21], particularly when appropriate action falls outside their remit or capacity [5, 22].

This study sought to fill this gap by documenting the challenges faced by RAs embedded within a community-based novel housing intervention. Because these challenges are multifaceted and often embedded in daily field interactions, addressing the study aim required supplementing the primary interview data with additional sources, including case studies, and observation notes. The main aim of this study was thus to explore the perceived impact of these challenges on RAs’ wellbeing and work, and the approaches they adopted in response—particularly when confronted with sensitive issues.

## Materials and methods

### Study setting and context

**Fig. 1:**
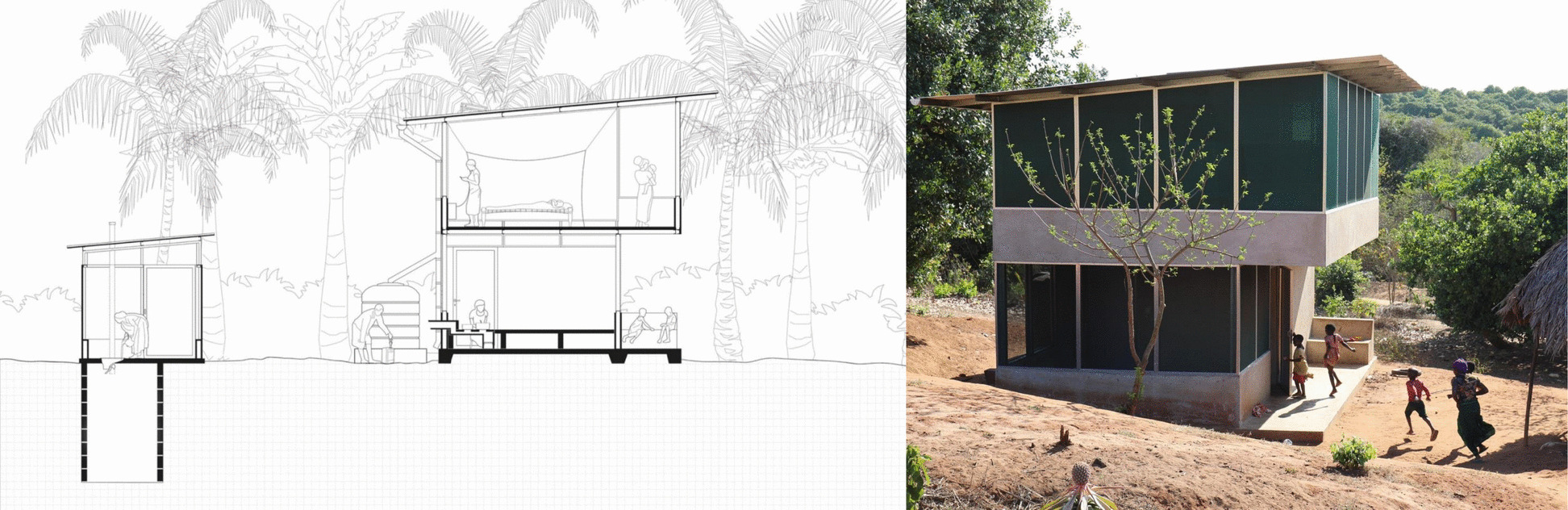
Star Home, a novel design home to provide comfort and better health

The current study took place in Mtwara region of southern Tanzania, within a novel housing intervention called *Star Homes* (Fig. 1). Part of the *Star Homes* intervention was a randomised trial evaluating the effectiveness of 110 novel houses, built across 60 villages, in preventing malaria, diarrhea, and respiratory illnesses among resident children under 13 years of age [23–25]. As part of *Star Homes’* research and implementation procedures, RAs were employed by a local host organisation based in Mtwara to assist in the implementation of the project. During implementation, RAs frequently communicated with residents of both *Star Home* and ‘control’ houses as part of routine data collection.

The population of the Mtwara region in southern Tanzania is predominantly composed of three major ethnic groups: the *Makonde*, the *Yao*, and the *Makua*. Within the study villages involved in the *Star Homes* project, the Makonde constitute the dominant group. The Makonde differ in various cultural aspects, including belief systems, social organization, and customary practices, from other ethnic groups in Tanzania. Like many coastal communities along East Africa, the Makonde are primarily Muslim, although a minority identify as Christian or adhere to traditional religious systems involving animistic forms of ancestor veneration. Historically, the Makonde are a matrilineal society, meaning that lineage, inheritance, and social affiliation are traced through the mother. As part of this system, husbands traditionally relocate to the villages of their wives after marriage. The primary language spoken is *ChiMakonde* (or Makonde), although *Kiswahili* (or Swahili), Tanzania’s national and official language, is also widely spoken.

The Yao and Makua constitute a smaller proportion of the population in the study villages compared to the Makonde. The Yao predominantly reside in areas bordering northern Mozambique and southern Tanzania and are widely known for their longstanding involvement in regional trade networks. Most Yao identify as Muslim, and ChiYao is commonly spoken alongside Kiswahili. The Makua trace cultural and linguistic ties to northern Mozambique. Makua communities are diverse in religious affiliation, including Muslim and Christian populations, and commonly speak ChiMakua in addition to Kiswahili.

### Design

This was a qualitative study incorporating elements of ethnographic methods, including participant observation and narrative case studies, such as investigators’ diaries based on field visits [26]. Participant observation notes were collected by BA, and narrative case studies were collected by SH. The study primarily built on key informant interviews that collected data using semi-structured interview guides and followed the Consolidated Criteria for Reporting Qualitative Studies (COREQ) guideline (Supplementary File 1).

### Participants

Respondents were purposively selected based on their knowledge of the challenges facing them in their routine work in the research communities. Participants in the field notes were pseudo-anonymised to conceal their identities. All RAs invited agreed to participate in the interviews, and no repeat interviews were required.

### Data collection

Qualitative interviews took place in participants’ workplaces and were based on a comprehensive interview guide (Supplementary File 2) co-developed with *Star Homes* staff and social scientists: JM, BA and FV. The interview guide was discussed and piloted among key staff and refined further to ultimately include questions about challenges commonly faced by RAs (at both office and community levels), common concerns arising within the community (e.g. family disputes, poverty, food, lifestyle), and specific concerns related to children’s health, including child abuse and neglect. Interviews lasted between 45 and 120 min and were conducted in Kiswahili by JM, a Tanzanian social scientist who had previously led a series of social science studies within *Star Homes*. All conversations were audio-recorded following written informed consent from participants. In addition, this study integrated the case studies (n = 31) collected by the research assistant (SH) with observation field notes (n = 16) collected by BA. Case studies documented specific issues from the community (e.g. family disputes, CSA), while observation notes broadly captured the general atmosphere, informal discussions with study staff, and community members. By virtue of their descriptive nature, the case studies and observation notes were used to supplement findings from the qualitative interviews by adding detail and supporting verification, but were not subjected to formal analysis. Data collection ceased after interviewing all available RAs and reviewing all relevant case studies, as no new themes were emerging, indicating thematic saturation [27].

### Data management and analysis

All audio-recorded interviews were transcribed and translated into both Kiswahili and English to ensure a transparent juxtaposition of the original quotes and their translations. The dual-language transcription helped preserve the concepts and nuances expressed in Kiswahili and minimised the risk of meaning being lost in translation. Transcripts were then analysed using Braun and Clarke’s (2006) six-phase approach [28] to thematic analysis using NVivo, a qualitative data management software.

First, all transcripts underwent line-by-line coding led by BA, allowing codes to be generated directly from the content of the KIIs. Initial coding of the first few transcripts produced a set of emergent codes reflecting meaningful patterns, ideas, and recurring issues articulated by participants. As coding progressed, codes were iteratively refined, merged, or expanded in response to newly encountered data and evolving interpretations (Supplementary File 3). Patterns across codes were then examined for conceptual relationships by BA, LvS, JM, and FV, enabling the construction of broader candidate themes in subsequent phases of the analysis. Following the completion of the coding, the landscape of the codes, in terms of their distribution, frequency and relevance to the research question was discussed among the researchers (SM, LvS, BA, and FV). Based on the discussion, the major and minor themes were further refined through interpretative analysis of the quotes, alongside case studies and observation notes [29].

### Ethical approval

The study was approved by the National Research Ethics Committee of Tanzania as part of the main Star Homes study on 17 June 2021 (reference NIMR/HQ/R.8a/Vol.IX/3695) and the Oxford Tropical Research Ethics Committee on 2 July 2020 (reference #533–20). The approval was renewed on 16 June 2025. All respondents provided written informed consent to participate in the study.

## Results

Participants included all research assistants working at the *Star Homes* interventions, the majority (94%; 15/16) were under 40 years (Table 1). Most (63%) were male, and majority (94%; 15/16) had college or higher-level education.

**Table 1:** Socio-demographics of the participants

	**Total**	**Percentage (%)**
Age		
20–29	8	50%
30–39	7	44%
40–49	1	6%
*Total*	***16***	***100%***
Gender		
Females	6	37%
Male	10	63%
*Total*	***16***	***100%***
Education		
University	5	31%
College	10	63%
Secondary Education	1	6%
*Total*	***16***	***100%***
Category		
Star Homes Staff	16	100%
*Total*	***16***	***100%***
Duration in District/Project		
1–2 years	0	0%
3–4 years	15	94%
5 years +	1	6%
*Total*	***16***	***100%***

**Fig. 2:**
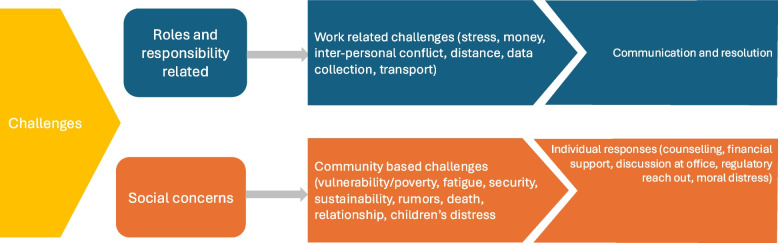
Challenges faced by research assistants

Participants broadly shared two categories of challenges (Fig. 2); one related to their roles and responsibilities within the work environment, and a second related to the community, where they regularly witnessed the challenges faced by the community members. Most challenges related to their roles and responsibilities ranged from work-related activities, including travelling long distances, to minor interpersonal conflicts. The challenges related to the study community varied from the vulnerability of community members to witnessing death, rumours, navigating their relationship with community members, and, most notably, the distress faced by children. RAs experienced a wide range of these challenges, including responding to personal and organization levels.

### Roles and responsibility related challenges

RAs expressed challenges related to their general role. For example, disruptions in data transfer and remote access to the central server due to internet outages were commonly cited sources of irritation. At times, these disruptions resulted in RAs having to re-collect data, further amplifying their frustrations.

*Yes, you go to a participant, collect their age, bring it to the data managers, then next week they ask you to go back to the village and collect the age again. You go to the same person from whom you already got their birth certificate the first time, they look annoyed but give it to you again, and you bring the age back to the office. After two weeks, the office tells you to go back and collect the age from the same participant. They are upset, angry, and you can tell they’re annoyed. IDI11*

Due to the wide distribution of research sites across 60 villages, research assistants often had to travel long distances, including up to 200 km by motorbike to reach the most remote locations. These travel demands became physically exhausting over the course of the project.

*If you look at the project’s strategy, the area coverage is a bit large, so the biggest challenge I’ve faced is the distance in reaching my participants. This was something I had to get used to. At first, it was easy because I had energy throughout the time. When we started follow-ups for ACD [active case detection] in 2022, my energy was high and it was exciting. I didn’t have experience riding a motorcycle, but from the first day I was given transportation, I gained confidence in using the motorcycle as a means of transport, driving almost 140 to 200 kilometers per day for data collection. It has been both a good and tough experience. We have been doing this for 3 consecutive years. IDI10*

Inherent within travel-related challenges were occasional breakdowns of the motorbikes, running over cats, dogs and other animals, driving through rough and muddy roads, and road traffic accidents.

Although most interpersonal conflicts among staff were minor, often stemming from simple misunderstandings and temporary in nature, a few of the incidents required staff meetings and resolution. Some of these issues were related to data collection, and equipment functioning, which would affect the quality of the data.

Other challenges were the hardships of travel to the study villages, leading to the development of pain, fatigue, accidents, and illnesses that impeded the flow of their work.

RAs further expressed concerns about what they perceived as insufficient training to handle community-based encounters involving psychological distress:

*Okay, I could have been taught how to deal with victims, at least taught a bit, especially how to handle a victim in the initial stages, like through isolation or if it’s depression, like how to deal with mental issues. I could have been taught those things because I am sensitive to them, and since I was a child, I prioritize understanding these things. But now I need education on how to deal with this. I’ve realized that it takes education from a distance because there are things I, as I am, might not be able to approach correctly without enough knowledge about the issue.* IDI13

### Community-based concerns

Outside of factors directly related to their working conditions, participants also expressed a range of challenges associated with the emotionally taxing nature of engaging closely with families affected by extreme poverty. Specifically, their work often placed them in direct contact with households struggling to meet basic needs, including food, shelter, and healthcare. This constant exposure served as a stark and painful reminder of the harsh realities many families face daily. What RAs found most distressing and unanticipated was the high incidence and severity of child maltreatment that surfaced during their fieldwork. Many spoke about the emotional burden of witnessing physical abuse, neglect, and exploitation as well as hearing disclosures of abuse.

### Poverty

Participants reported encountering communities in desperate need of money to buy food, clothes, and basic amenities, with high rates of poverty common among *Star Home* participants. When faced with a sick community member not included in the study, they felt they could not simply advise them to go to the hospital and then leave without being affected: *“if someone needed money to go to the hospital or to buy food, and I couldn’t help, I reflect on that a lot”* (IDI15). This was particularly the case for the RAs who were also trained health workers, who felt a sense of moral duty to transport patients to a health centre.

RAs recounted encountering distressing situations as part of their work. In one instance, a team of RAs were asked for help early in the morning, while on their way to the study villages:

It was around 5:45 AM when we were on the road, having passed through some villages of our colleagues, like X and Y. Along the way, we encountered people on motorcycles bodabodas carrying someone and they were alerting us to stop [.…] We then inquired about the person they were carrying, and they revealed that he had passed away. It was very distressing. They had collected the body from a hospital in Nyangamawe, which is very far from the town. They were transporting the body to their home for burial but had run out of fuel. They explained they couldn't afford to hire a vehicle, so they decided to leave early in the morning to avoid encountering the police, as transporting a body on a motorcycle is against the law. IDI14

### Child maltreatment

Multiple participants raised concerns related to children’s welfare in the study villages. Participants reported being distressed by the way children were disciplined, both verbally and physically, and truancy was a prominent concern for RAs, who often found children from participating households roaming around the houses on weekdays. When asked, parents reported that the decision of whether to attend school was largely left to their children. Witnessing this left some participants disappointed:

The first event is children dropping out of school at a young age, while living with both parents. But you find that the parent refuses to take any responsibility. When you visit as a researcher, you ask what the issue is, and the father doesn’t have time to give you sufficient information, he just refuses. It stresses me out when a child leaves school at six years old, and the parent is there. How does a parent let their child leave school at such a young age? You’re eating from the same bowl at mealtime, and you don’t seem to care, no emphasis [on attending school]. Maybe I went to talk to the head teacher, but there was no action. This happens a lot in Mtwara. IDI11

Child marriage often occurred when girls eloped to evade family surveillance. Such elopements also appeared to be driven by poverty and food scarcity. Some RAs observed high rates of divorce in these communities. While some believed these practices to be embedded in local traditions and cultural norms, others viewed them as a consequence of inadequate parenting and limited educational opportunities.

Well, forced marriage isn’t a big issue because, like I said, children here make their own decisions. A 12-year-old girl might leave school after grade six and go live with a man in another village. She does it willingly, not because she’s forced. But eventually, she might come back pregnant. Then the issue is either the man who got her pregnant has to come and marry her, or the parents start asking who this man is. Sometimes the parents don’t take it seriously until it’s too late. But generally, it’s not about force. The children often leave home and go live with a man on their own. IDI16

Although education, parenting, and neglect varied across households, the widespread pattern of children leaving with older men, subsequently becoming pregnant, and parents settling the matter through negotiation and marriage was common.

### Child sexual assault

Despite the range of challenges in the community, all RAs responded that they were most disturbed by the stories and incidents of child sexual assault (CSA) encountered through their work, particularly when cases involved *Star Home* beneficiaries as either victims or perpetrators of CSA. Indeed, CSA was also cited as a reason for the high rates of truancy:

*There was a child who dropped out of school in Form One, saying the issue was the teacher, who was pressuring her to have a sexual relationship with him. Another child dropped out in Form Two for the same reason – that teacher wanted to sleep with them. IDI12*

Extreme poverty, according to participants, was putting children at high risk of exploitation. For example, participants commonly described hearing about girls who had been forced into early marriage, some as young as 8 or 9 years old. When asked why they were married at such a young age, participants mentioned the high burden of rearing children in the villages, including having to provide food, clothes, and education. Another participant described a case where a six-year-old girl and her friend had been raped by the girl’s uncle. The girl’s father then took his child to the hospital to confirm the assault, and took a letter to the village chairperson, asking them to report it to the main police station. At the time that the participant was learning of this, the chairperson had yet to report the incident to the police as the chairperson ‘*did not have bus fare*’. RAs felt devastated to learn the level of vulnerability. Other participants recounted that where the victim and perpetrator were part of the same family, the release of the perpetrator was sometimes considered essential for the survival of the family.

RAs also voiced deep frustration and concern over harmful cultural norms and practices in the villages that systematically failed to protect children who had experienced CSA. One participant learned of a case of incest involving a father and his 11-year-old daughter. Despite the case having been reported to the local authorities, the child’s mother ultimately chose not to press charges for fear of being blamed by his family members for the incarceration of her husband. According to the participant, the child’s mother also faced isolation from her own relatives and neighbours for reporting the assault to relevant authorities. This fear of ruining the family structure or relationships with other relatives was referred to as violating ‘*muhali’* and was reportedly the reason why alleged incidences of CSA by family members and relatives made known to participants were not reported or prosecuted. One notable exception, however, was CSA reported or perpetrated by teachers, as their origins from outside of the family or relatives meant that they were not protected by *muhali.* Other cultural practices of ‘*jando*’ and ‘*unyago*’, initiation rites for male and female children, respectively, where boys are circumcised and females are taught on preparation for marriages, contributed to an accepted risk of CSA.

*We have initiation rituals (Jando and Unyago), where young children are taken to be prepared for marriage. When you ask why they do this to young children, they say it’s just preparation for marriage.* IDI1

Participants reported that some community members held the belief that a sex with a child, including infants and toddlers, is auspicious in terms of money and fortune, *“It’s just habitual, and some people believe in superstitions that if they do something cruel to a child, it might somehow improve their own life” IDI5.*

Respondents also shared their frustrations over the impunity granted to perpetrators by village leadership, which also extended to government authority level. This was particularly true in the case of CSA occurring in the family, where "*we found that these matters are treated with family ties being valued over the child’s justice”* (IDI1)*.* Participant cited examples of elected village chairpersons reluctant to confront the perpetrator, often because such cases were immediately negotiated by the family members and accusations were withdrawn. As a result, the perpetrators were often never taken into custody, or, if so, were released prematurely, and cases did not advance to court. Participants reiterated that failure of prosecution was not due to an absence of legislation, but rather voiced their frustration with a ‘lack of implementation of 30-year-old CSA laws’:

The challenge that makes things like this happen is that if people were aware of the consequences these individuals face later due to these actions, I think people would take it more seriously. If social welfare took it more seriously, even we, those of us in normal positions, would say that social welfare is doing their job properly, and in that case, I would be confident that if I took my case to social welfare, action would be taken. So, the problem starts from the top, making it difficult for those of us at the bottom to make any decisions. IDI10

Another participant recounted a particularly egregious case involving a two-year-old girl and her sixteen-year-old uncle. Despite the child’s parents having taken their child to the health centre to confirm the rape, and a reporting of the incident to the Village and Ward Executive Officer, which eventually reached a social welfare officer, a failure of the judicial system ultimately led to the release of the alleged perpetrator:

*[The child’s] parents received a call from their village chairman who asked them to appear in court two days later. They walked all the way from the village to the city. After arriving, they found the case had been already read, and the suspect was released. The suspect and his clan were told that they had to return to the police station two days later to witness the suspect being whipped for his crime.* Field Notes-III

### Impact on research assistants

It has affected me a lot…The most stress comes from witnessing the abuse of children… I don’t even know how to explain it; it has affected me to the point where I feel like a victim. IDI9.

Research assistants felt the greatest impact when witnessing or hearing about cases of child maltreatment, particularly those involving CSA. The effects of encountering such challenges ranged from feelings of helplessness, frustration, and anger to severe psychological distress. Incidents of CSA were especially distressing, and some RAs internalised the experiences so deeply that they began to see themselves as victims.

RAs expressed profound discomfort related to the vulnerability of children who were neglected or did not receive proper care*,* felt vulnerable when exposed to conditions where they could not intervene*,* and felt helpless when parents were not receptive to their advice and requests for additional care.

As a human, it affects me deeply because these are young children, vulnerable and helpless. When a child is born, they rely so much on their parents for protection, and when the very person who should safeguard them doesn’t take their upbringing seriously, it troubles me deeply, yet there’s not much I can do. If you try to intervene, the parents don’t cooperate. IDI1

Some of the RAs also expressed intense frustration and anger, particularly toward the perpetrators. Their encounters with these cases evoked deep emotional turmoil, especially as they saw the perpetrators moving freely in the community without accountability. The sense of injustice and helplessness brought on by witnessing the suffering of children who lacked the means to protect themselves was deeply unsettling. The victimisation of vulnerable children, coupled with the perceived impunity of the offenders, provoked strong emotional responses.

The thing is, I could hit him with something, even to the point of death. That’s why I’m saying the truth, because people like him should not exist in society. They are animals. They don’t deserve to live like normal human beings…… Yes, that’s… as I speak here, I even feel like crying. These incidents really hurt, those children are small, they don’t know anything, they can’t protect themselves, and the family itself is poor. You do something like this to a child, really? They don’t know anything, and they’re taken to the hospital. Do you really consider yourself a normal human being? IDI6

Most of the research assistants carried home a deep sense of frustration, weighed down by the helplessness of the victims they encountered. Many voiced a strong desire to report the perpetrators and pursue justice but felt restrained by the boundaries of their professional responsibilities, which prevented them from acting on these impulses.

*I feel bad and sometimes wish I could arrest the person myself and take them to the police station so it could be a lesson for others committing such acts or those who fail to report these incidents. IDI5*

### Responding to challenges

Different approaches were used by participants to respond to these two categorical challenges (Fig. 2).

In the case of work-related challenges, and although some inter-personal conflicts emerged from misunderstanding and poor communication, participants identified a horizontal leadership structure as key to addressing these types of challenges:

Our director likes to use the term "horizontal management." … If you have something, or an issue, you go to xxx, yyy, zzz, or anyone else who can help solve the problem. They don’t try to show off that they are managing. Yes, we know that mostly they are managing us, but there’s no attitude of trying to show that "I’m the director, I’m in finance, I’m here…" There’s none of that.. IDI9

When it came to work-related challenges, RAs appreciated the weekly meetings, where RAs could voice their concerns and find collective solutions. None of the participants felt they had to restrain themselves when faced with problems, allowing open and unrestricted communication with the leadership structure within the office.

Responding to community-based challenges, however, was reportedly more complicated. Over time, some RAs developed a moral responsibility towards their beneficiaries. Obligations were particularly felt towards children and spanned concerns related to various aspects of their welfare (health, neglect, education, punishment and workload). In response, RAs often used their own money or advocated on behalf of children for additional resources, such as school bags and uniforms:

The next week, I went to check her progress. If she hadn’t been allowed to go back, I was prepared to leave my bag and go to school myself. The headmaster understood the situation and allowed her to continue studying. She was provided with food and attended school regularly. I kept asking her how things were going, and she would say, "I don’t have notebooks," so I would give her money to buy one. The next time she would say, "I need a pen," and I would give her money for that. She has now completed the seventh grade. Last year, she was in the sixth grade, and now she has finished the seventh grade. IDI11

RAs also pooled resources for their beneficiaries, coming together to assist the families from whom they were tasked with collecting data. Their readiness to help and offer support was driven by a deep sense of awareness of what was happening in the community. Community members affected by familial disunion, disability, hunger, and poverty were prioritised by the RAs for support with these pooled funds.

*When it comes to food and clothing, we often pool money and assist them; one of our members even has a house where there’s a mother who is blind, and her husband has another wife. When he leaves, he doesn’t always leave any resources, and I can’t tell if there’s really nothing or if she just doesn’t get anything. RA Paul often tells me, “I feel so bad for that mother – she goes hungry.” Sometimes, that husband takes a hoe, goes to the nearby farm, and works to earn some food. We provide her with some funds for essentials like rice, oil, and clothes. IDI1*

Apart from cases of poverty, and associated vulnerabilities in the communities, incidents of child sexual abuse drew prominent attention from the RAs. Their responses were often immediate and coordinated, particularly after discussions with peers and at the office. Nonetheless, these responses were short-lived and ultimately futile in the face of hesitancy among parents to lodge formal complaints against the perpetrators. When RAs attempted to support mothers of abused children, they were further frustrated by the mothers’ reluctance—or their families’ refusal—to pursue legal action.

I’m hesitant because we have tried empowering mothers whose children have been assaulted, but they remain unwilling. We assist with transport and access to legal aid, but they are unwilling. […] Sometimes, you may even think about taking the child away, but then you wonder what would justify that. IDI1

Others sought to achieve a balance between responding to cases of abuse and fulfilling their professional responsibilities. RAs offered suggestions to parents on averting the incidents for example, by taking children to the farm.

*It means everything should be done in balance. If you dwell too much on abused children, you'll fail to collect data. So, I've kept my mind focused because I’ve faced these challenges, played my part in assisting where I was, but then I return home and educate parents so that now they take their children to the farm. When I meet similar situations, I’ll provide education and ensure the parents understand, and after that, I keep my mind clear to continue with other things. IDI11*

That said, participants also felt that their responses were constrained by their professional roles and their awareness that getting too involved could inadvertently entangle them in the cases themselves. This, in turn, they felt could jeopardise the implementation of the study and affect how they were perceived in the community.

The work I do doesn’t allow me to be at the forefront because if community members learn that I or we, as Centre for Scientific Knowledge (CSK), are there following up on cases, they might start viewing us differently, and we could lose what we’re working toward. To address these issues effectively, you have to be proactive and use the existing authorities, like the village government, although they don’t follow up closely. IDI5

## Discussion

Commonly employed within global health research, RAs operate at the delicate interface between communities and research institutions, often working in a liminal space—frequently without the authority, resources, or power to act on the conditions they are exposed to in the community [22, 30]. Our findings suggest that RAs bear a double burden in fulfilling their professional roles. First, they face substantial logistical and environmental challenges, including extreme weather, difficult field conditions, long distances to field sites, and poor road infrastructure, all amid constant pressure to maintain research quality. Second, their empathy-driven engagement with vulnerable communities exposed them to poverty-related hardships, such as food insecurity, lack of clothing and education, and limited capacity to care for children [31]. Over time, RAs tend to develop close relationships with participants, exposing them to deeply distressing realities—including gross human rights violations, most notably child sexual abuse (CSA).

In this study, facing these harsh realities led to moral distress and posed an ethical dilemma for RAs over how to support victims and prevent further harm. RAs reported feeling powerless to intervene and emotionally consumed by incidents that were unexpected and beyond their role, highlighting a troubling sense of impunity for perpetrators and an urgent need for safeguarding measures. These experiences left many RAs with indicators of vicarious trauma, underscoring the importance of preparing research staff through training and institutional support to navigate such unanticipated and ethically fraught realities [30, 32].

### Challenges encountered

Of all the challenges encountered in their work, RAs were concerned with truancy and the consequences of missing out on education, early coercive marriages, and, most worryingly, CSA, as an under-recognised, under-appreciated, universal public health issue that negatively and permanently impacts child psychosocial development with life-long repercussions not only for the victims but also for family members and the community [33].

While CSA is largely rooted in poverty or structural violence, its socio-cultural dimensions should not be underestimated [34–36]. Instances of CSA in the current study appear to be partially fuelled by a belief that sexual relationships with very young children are auspicious. Consequently, attributing CSA in the Mtwara region exclusively to poverty would be an unfair reduction. In particular, due consideration should be given to socio-cultural beliefs surrounding CSA occurring within the family, including how such incidents are concealed and never formally reported to relevant authorities. Participants in the current study, for example, discussed the concept of *muhali,* which can be understood as avoiding family disputes, which allowed CSA to continue. The result is the child’s victimisation for the benefit of the family’s relationships, perpetuating a cultural environment that works against the prevention of CSA.

### Impacts and responses

RAs shared a range of impacts and responses based on the nature of the incidents they were exposed to. The greatest impact occurred when they learned about the cases of CSA. These cases evoked helplessness, frustration and anger including a sense of guilt for not being able to do more. Some developed coping mechanisms, such as taking feasible steps at the site, reporting to authorities (social welfare officers, police), and sensitising family members. Observing the futility of such acts, however, was debilitating for many RAs, yielding responses consistent with extant theories of empathy and moral distress.

Human empathy develops over a progressive sequence of initial affective arousal (vicarious response), followed by emotional understanding and ultimately the regulation of emotions in preparing a well meditated cognitive response [37]. Witnessing distress can trigger vicarious responses, in which the observer affectively approximates another’s experience, as a fundamental human behavioural response [38]. Human reactions to witnessing cruel acts of violence often trigger stress responses, which are amplified when the victim is particularly vulnerable, such as a child [39, 40]. Subsequently human reactions can trigger a temptation to redress the perceived injustice [41]. While affective reactions can impact the well-being of a witness, responses that call for redressing the observed harm can also lead to frustration, anger and deep disappointment, or ‘moral distress’ [42]. Specifically, moral distress occurs when one knows the ethically correct action but is constrained from taking it or feels powerless to take that action [43]. While the universality of empathy among most humans does evoke both visceral and cognitive responses, they can vary at an individual level, with some being extremely concerned and some less so [44].

### Implications

RAs can find themselves in a dilemma about whether, how, and to what extent to immerse themselves in communities targeted by global health research interventions [45]. This dilemma can lead to moral distress among RAs who face challenging and difficult cases of human rights violations and a desire to redress the source of stress in their communities. Researchers, institutions and funding bodies implementing research projects in LMICs should have a continued awareness of the context, including vigilance regarding the integrity and well-being of research assistants alongside the study participants [45, 46]. Consequently, senior researchers should implement training and field-based safeguards to protect RAs from psychosocial trauma. These may include providing regular mental health and psychosocial support, regular supportive supervision, access to counselling, and practical guidance for how to manage distressing or traumatic experiences [47, 48]. Traumatic cases, such as those arising from household violence and child abuse, even if they occur occasionally, can have a profound impact on RAs. Therefore, training should include guidance on how to handle such cases through teamwork and collaboration with community members, village leadership, social welfare officers, and law enforcement agencies. Additional studies are critical to explore the social, cultural and familial practices conducive to CSA, including the actions that can be taken to thwart such practices.

### Strengths and limitations

This study calls attention to the numerous challenges faced by RAs, as individuals commonly employed within community-based global health research. The current study is not without limitations, however. Firstly, emerging concerns were captured by interviewing field staff and not directly interviewing the community members (victims or their parents and the perpetrator), which could have offered first-hand accounts of the issues. Nonetheless, as part of the four-year study, the RAs were immersed in the community, with substantial familiarity with the local social, and cultural context. Second, this study does not attempt to capture the burden of CSA at the study site; therefore, the small number of CSA cases reported by RAs may appear accentuated because the focus is on the qualitative aspects of these cases, such as their impacts on and the responses of RAs. As much as these sensitive topics may have caused distress to RAs, they may also have been influenced by the social taboos, potentially triggering social desirability bias and leading to a greater emphasis on CSA. Third, although the case studies and field notes enriched the study, the limited diversity of respondents restricted opportunities for triangulation. Fourth, the study findings are grounded in the specific communities of Mtwara and thus cannot be generalised to other contexts, nor are they immune to the temporal dynamics of these communities. In addition, the data may have been affected by selection bias due to the specific type of respondents and their cohorts within the study. Finally, interpretative analysis in this study may have been shaped by the researchers’ positionality and prior assumptions.

## Conclusions

RAs employed in community-based research rarely receive formal training to support them in navigating the ethical challenges they may encounter in their roles. The present study indicates that RAs experienced particular distress in relation to children’s well-being, particularly in cases involving sexual abuse. For some RAs, moral distress arose from being unable to intervene or offer appropriate support. These findings underscore the need for urgent action through a combination of top-down and bottom-up approaches. Specifically, community-based studies should proactively anticipate the potential occurrence of distressing and co-occurring events (including CSA) and embed community engagement strategies that facilitate clear, structured, communication between communities and relevant authorities. Furthermore, mechanisms that promote open discussion of research and community-related challenges within study teams and among relevant stakeholders may help mitigate the risk of moral distress among RAs. To this end, RAs should be adequately prepared through targeted training and supported with appropriate resources to respond to such situations and manage their associated emotional burden.

## Supplementary Information

Supplementary Material 1. COREQ: COnsolidated criteria for REporting Qualitative research.

Supplementary Material 2. Interview guide adapted for the study.

Supplementary Material 3. Coding framework (codebook).

## Data Availability

Because of the nature of the qualitative data in this study, even if the data are anonymized, potential respondents are identifiable. Both MORU and local ethics committee restricts the sharing of data that can potentially identify the respondents. The data is available upon request to the Mahidol Oxford Tropical Medicine Research Unit Data Access Committee (datasharing@tropmedres.ac) complying with the data access policy on case-by-case analysis ([https://www.tropmedres.ac/units/moru-bangkok/bioethics-engagement/data-sharing/moru-tropical-network-policy-on-sharing-data-and-other-outputs](https:/www.tropmedres.ac/units/moru-bangkok/bioethics-engagement/data-sharing/moru-tropical-network-policy-on-sharing-data-and-other-outputs)).

## Abbreviations

CSA: Child Sexual Abuse
KII: Key Informant Interviews
RA: Research Assistant
