# Supplementary material for: “The most stress comes from witnessing the abuse of children” —challenges faced by research assistants in community-based research in Mtwara, Tanzania
Source: BMC Public Health. 2026 Feb 7;26:864. doi: 10.1186/s12889-026-26485-3 (PMC12977482; doi:10.1186/s12889-026-26485-3)
Supplement: Supplementary file 2 — Supplementary Material 2. Interview guide adapted for the study. [file 12889_2026_26485_MOESM2_ESM.pdf]

**STUDY TITLE:** STAR HOMES PROJECT **COMPONENT**: SOCIAL SCIENCE
**DATA COLLECTION GUIDE TYPE:** GBV IDI GUIDE – Oct-Nov2024

## A: STUDY-SPECIFIC DETAILS

INTERVIEW DATE: |_|_|/|_|_|/|_|_|_|_| SS INTERVIEWER INITIALS: |__|__|__|

RESPONDENT CATEGORY: STAR HOMES STAFF **START TIME: |__|__|:|__|__| am/pm**

## B: INFORMED CONSENT PROCEDURES

**Remind the respondent about the use of an audio recorder and turn it ON now!**

## C: SOCIO-DEMOGRAPHIC DETAILS

| | |
|---|---|
| **Unique ID No.** | |
| **Age** | |
| **Sex** | |
| **Education level** | None |_|; Primary |_|; Secondary|_|; High school|_|; Tertiary (Collage / University) |_|; Other |_| specify___________________ |
| **Professional background/Cadre (Dr, CO, AMO, Nurse, Lab technician etc)** | |
| **Total years of experience** | |
| **Years of experience at Star Homes** | |

## D: THE INTERVIEW

| TOPIC/THEME | SUGGESTED QUESTIONS AND PROBES |
|---|---|
| **CONSENT CONFIRMATION** | Have you consented to participate in this interview? Yes/No (*If no, end here*) |

| | |
|---|---|
| **WARM UP/GENERAL QUESTIONS** | **1)** I am glad we have this moment to talk, how are you today? How about your family?<br><br>**2)** Do you have children? How many? How old are they?<br><br>**3)** Are you originally from here? If not, where if you won't mind?<br><br>**4)** How long have you lived in Mtwara region?<br><br>**5)** Can you tell me about your experience of working with Star Homes project? How has it been?<br><br>**6)** Any particular incidents that you like? **Probe**: any incidents or matter from office, field?)<br><br>**7)** Any particular incidents that you dislike? **Probe**: any incidents or matter from office, field?)<br><br>**8)** Let's focus on something that has affected you, tell me about any incidents that you could recall from the study villages/area? **Probe**: cases of family disputes? Poverty, Health issues, Fights, physical violence? jealousy? violence against children?) |
| **VIOLENCE AGAINST CHILDREN IN THE STAR HOMES PROJECT AREA** | **9)** Over the period of time, you have worked in the village, what incidents related to violence against children have you heard within the participating families? **Probe**: child sexual abuse, acts, incidents, violence? forced/early marriage, school attendance, workload, child labor<br><br>**10)** Please explain to me further about each incident. **Probe** for narration by the research assistant<br><br>**11)** Have you heard of such incidents before? **Probe**: Outside the study villages (areas), outside the research participants? If so, please tell me |

| | |
|---|---|
| | how they are different.<br><br>**12)** From your opinion, does the violence against children in study area resemble anywhere else? Please, tell me more about that. |
| **VULNERABILITY OF RESEARCH ASSISTANTS** | **13)** After hearing of these (GBV) incidents, how did you react? **Probe**: consequences of reactions by the interviewee<br><br>**14)** Did you ever attempt to respond to such incidents in the study villages?<br><br>**15)** Did you feel any power dynamics or obstacle to respond to such incidents? **Probe**: with families, local authorities, organizational limitations?<br><br>**16)** Having you here today, how were you able to cope after hearing such incidents? **Probe**: What happened to you? What mechanism did you adapt to cope up? How did you perceive/interpret such incidents?<br><br>**17)** What resources or support systems were available to help you deal with these situations?<br><br>**18)** Did you feel prepared or trained to address child sex abuse during fieldwork? **Probe:** Were there any gaps in support that you wish had been addressed?<br><br>**19)** Did you feel that your concerns were taken seriously by your supervisors? Can you explain on it?<br><br>**20)** Were you ever worried about backlash or consequences for reporting such incidents?<br><br>**21)** What impacts did you experience from hearing these incidents? **Probe**: *physical* - feeling weak, lethargic, exhausted, *emotional* - mental, spiritual, long term, short term. *Sleeping disturbance* - anxiety, low mood, guilt, regret, revenge, self-loathe<br><br>**22)** What did you do to alleviate discomfort from hearing such incidents? **Probe**: inability to help, inability to support, inability to change the situation |
| **RECOMMENDATIONS ON COPING AND FUTURE RESEARCH** | **23)** Do you feel that you were vulnerable (helpless) when you heard such incidents? Can you explain how?<br><br>**24)** What solutions do you think are there to tackle such problems? |

| | |
|---|---|
| | (**Probe**: for the problems of violence against children and your vulnerability, solutions to field workers' problems/dilemma)<br>**25)** If it was not a research project environment, how would you have reacted to such incidents?<br>**26)** How would you recommend research site leadership to tackle such problems in future?<br>**27)** Any additional thoughts about such incidents (free thoughts) |
| **CLOSING** | **28)** What else that we have not talked about that you would like to share with me? Probe: Anything else about the Star Homes Project?<br>**29)** Do you have any questions?<br>**Interviewer**: Give an overall summary of the talk and thank the respondent for his/her time. |

**RECORDER TIME: |________________________|** **END TIME: |__|__|:|__|__| am/pm**
