# Supplementary material for: “The most stress comes from witnessing the abuse of children” —challenges faced by research assistants in community-based research in Mtwara, Tanzania
Source: BMC Public Health. 2026 Feb 7;26:864. doi: 10.1186/s12889-026-26485-3 (PMC12977482; doi:10.1186/s12889-026-26485-3)
Supplement: Supplementary file 3 — Supplementary Material 3. Coding framework (codebook). [file 12889_2026_26485_MOESM3_ESM.pdf]

Child sexual abuse focused empirical research

Codes

Name	Description	Sources	References
Challenges	Challenges shared by respondents not necessarily related to CSA	1	2
Data collection	Data collection related challenges	12	46
Distance	Distance from office/residence to study sites	7	11
Inter-personal conflict	Conflicts between staff affecting their work	5	15
Money	Challenges related to money	5	6
Stress	Stress in general and affecting the work	9	22
Vulnerability of study participants	Vulnerability of study participants that affects field workers	9	39
Child education	Concerns related to child's education	15	120
Child Health	Child health related challenges and issues	12	18
Child labor	Child labor	12	26
Child marriage	Marriage of children	13	45
Child punishment	Various kinds of punishment to child (physical, verbal)	12	44
FAMILY LEVEL	Family level risk factor of CSA	1	4
Family	Family members as risk factor of CSA	15	83
Parental supervision	Parental supervision on CSA	8	30
Relatives	CSA within the relatives	9	36
INDIVIDUAL LEVEL	Individual level risk factor of CSA	1	1

Name	Description	Sources	References
Vulnerability	Vulnerability emerging from various circumstances	11	39
INSTITUTIONAL LEVEL	Institutional level risk factor of CSA	8	14
Moral distress	Moral distress among field workers who are exposed on CSA	8	39
Dilemma	Dilemma of field workers	9	17
Situated learning	Learning based on the situation or informed by the situation.	4	12
Neglect	Child neglect	6	14
POLICY LEVEL	Policy level risk factor of CSA	0	0
Regulatory	Regulatory processes, challenges affecting CSA	16	79
Relationship	Relationship with community members	13	43
Appreciation	Appreciation on staff's performance, and celebration	15	61
Death	Death of a community member/participants	3	3
PTSD	Post-traumatic stress experiences	8	41
Skills	Skills related to identification of CSA, processing and management	4	13
Training	Training related to how to cope up with CSA	12	23
Response to child's distress	Response to children's distress who were participants in the study	15	104
Roles	Roles of respondents	6	16
Fatigue	Exhaustion because of the roles and responsibilities	2	6
Security	Security working in the field	3	13
Rumours	Rumours related to Star Homes or participation	5	6
SOCIETAL LEVEL	Societal level risk factor of CSA	0	0
Culture	Culture, custom and tradition	13	85
Jando and Unyago	Cultural practice of preparing children towards adulthood teaching them sexual education and how to deal with the sexual acts.	2	2

Name	Description	Sources	References
Perpetrator	Characteristics of a perpetrator who is unruly and dangerous	5	8
Village leadership	Leaders or authorities of the village who could act on CSA	11	43
Sustainability	Sustainability of the project	7	13
